# Emerging from the Shadows: Intrinsic and Extrinsic Factors Facing Community Health Workers in Western Cape, South Africa

**DOI:** 10.3390/ijerph17093199

**Published:** 2020-05-04

**Authors:** Wilson Majee, Laura Schopp, Levona Johnson, Adaobi Anakwe, Anthea Rhoda, Jose Frantz

**Affiliations:** 1 Department of Health Sciences and Public Health, University of Missouri, Columbia, MO 65211, USA; 2 Department of Occupational Therapy, Faculty of Community and Health Science, University of the Western Cape, Western Cape, Bellville 7535, South Africa; 3 Department of Health Psychology, University of Missouri, Columbia, MO 65211, USA; schoppl@health.missouri.edu; 4 Department of Physiotherapy, Faculty of Community and Health Science, University of the Western Cape, Western Cape, Bellville 7535, South Africa; levjohns@gmail.com (L.J.); arhoda@uwc.ac.za (A.R.); jfrantz@uwc.ac.za (J.F.); 5 Master of Public Health Program, University of Missouri, Columbia, MO 65211, USA; adaobi.anakwe@slu.edu

**Keywords:** community health workers, intrinsic, extrinsic, challenges, health care services, rural

## Abstract

Community health workers (CHWs) have been identified as a key component of the health workforce in South Africa. However, the efficacy of CHW programs continues to be limited by a poor understanding of facilitators and barriers to CHW engagement. This study explores intrinsic and extrinsic factors that CHWs face. We conducted in-depth interviews with 20 CHWs in order to understand the challenges they may face as they implement their duties linked to the primary health care strategy in the Western Cape, South Africa. All interviews were audiotaped, transcribed verbatim, coded and analyzed using NVivo 12. Drawing on narratives of CHWs, we illustrate the complex issues surrounding CHW outreach in poor rural communities. The CHWs identified five key areas of challenges with respect to personal health, gender issues, poor community understanding of CHWs roles, environmental challenges and lack of patient adherence. These all hinder the ability of CHWs to meet their personal and familial needs, as well as those of the community members they support. There is a need to address the intrinsic needs of CHWs in order to ensure their emotional and physical well-being, as well as a need to create an awareness of the roles of CHWs.

## 1. Introduction

Ensuring a healthy population is one of the essential goals of any government. Globally, governments have recognized the importance of providing primary health care at community levels. In 2010, South Africa implemented strategies to transform the health system from an individualized, passive, curative, vertical system to a population-based, integrated, proactive model [1]. Because of this proactive approach, community health workers (CHWs) have increasingly become central to the “re-engineering” of health care services. CHWs are lay people who live and work almost exclusively in the communities they serve. They perform multiple functions, including patient and community education, health service provision, decreasing cultural, linguistic and literacy barriers, linking people with community resources, and facilitating patient communication and adherence to care [2]. Because they work in their own communities, CHWs are knowledgeable about the socio-cultural context in which health services are received and health behaviors occur [3,4]. Qualitative reviews and studies in South Africa reveal that that CHWs increasingly provide health services and health-promoting activities [3] and excel at identifying community problem because of their local connection to the community [5]. In 2010, South Africa’s national Department of Health launched the Re-engineering Primary Health Care initiative that utilizes CHWs to support a preventive and health-promoting strategy for primary health care [6,7]. In this model, CHWs in South Africa provide integrated health and social care to households and form the “links” and “bridges” between health and social care providers and poor communities [7]. Although the value and impact of CHWs in preventing and managing a variety of chronic diseases, especially in low- and middle-income countries, are acknowledged [7,8,9,10,11,12], the efficacy of CHW programs continues to be limited due to poor understanding of CHW needs, lack of institutional resources, poverty, and persistent health worker shortages [7]. For example, a study in South Africa demonstrated that the role of CHWs and their success as interventionists may depend on their own health status [13]. Despite decades of being used at the frontlines, CHWs remain in a grey area at the fringes of the health system, lacking adequate support, and are therefore unable to provide optimal care for their patients and themselves.

Previous studies suggest that CHWs are motivated by appreciation from managers, respect from patients and family members, and acquisition and sharing of health knowledge, but discouraged by low salary and poor working conditions, limited health supplies, lack of transportation, disrespect from other health professionals, and limited support from a rigid and hierarchical health system [14,15,16,17]. While these factors are broadly understood, their contextualization to inform program development or optimization has been weak. This study uses the Two-Factor theory to explore and illuminate factors that cause dissatisfaction among CHWs and thus assists in addressing them and in shifting attention towards motivational factors. The Two-Factor theory posits that certain factors in the workplace cause job satisfaction, while a separate set of factors cause dissatisfaction [18]. Factors that cause dissatisfaction in the workplace are extrinsic (or independent of the work itself) and are linked to things such as compensation, job security, working conditions, quality of leadership, and relationships among employees. On the other hand, factors for satisfaction are intrinsic conditions of the job itself and include responsibility, job satisfaction, recognition, achievement, opportunities for growth, and advancement [18]. Intrinsic factors tend to increase motivation when they are present, while extrinsic factors tend to reduce motivation when they are absent.

Understanding and addressing these issues will ensure that community health worker programs are effective and sustainable [19]. This study is part of a larger, ongoing research project conducted with rural CHWs in South Africa to understand (a) their motivation for participating in self-management training, (b) skills gained from training and (c) perceived impact of training on CHW health behavior, both personally and as health professionals. The focus of the research for this paper was based on a week-long self-management training in which CHWs learned goal setting and action planning skills. Self-management has been defined as collaborative effort between the individuals, families, and health care professionals to manage symptoms, treatments, lifestyle changes, and psychosocial, cultural, and spiritual consequences of health conditions [20]. Self-management training evaluation findings are reported in our earlier publication [13].

Although our semi-structured interview guide has a strong focus on the experiences and evaluation of the self-management training CHWs completed, intrinsic and extrinsic factors were repeatedly mentioned during interviews. This warranted an independent report of these secondary findings on an area that is both novel and under-researched.

## 2. Methods

### 2.1. Study Setting and Design

This qualitative study used a face-to-face semi-structured interview guide (Table 1) to explore the factors that impede CHWs’ ability to deliver services effectively. This study was conducted in two small rural communities in the Western Cape Province of South Africa, with a total population of 8443 (Greyton = 2780 and Genadendal = 5663) [21]. Both are rural communities with more than 70% of the population from the disadvantaged population groups in South Africa (“black” and “colored”) [21], and both have community health workers who play active roles in providing primary health care. CHWs in both communities serve a population with diverse chronic conditions including cancer, diabetes, HIV/AIDS, and stroke. Health resources available in these communities include two primary health care and mobile clinics (one in each town) and two satellite clinics (in Greyton). Provisions for pedestrian and bicycle travel are minimal [22]. These communities were purposively selected for their socio-economic profiles and CHW-driven health promotion.

### 2.2. Recruitment of Participants

Following approval from the institutional review boards of the University of Missouri (IRB # 2009691 HS) and the University of the Western Cape (EC 15/3/16), participants in this study were recruited and interviewed in March 2017. CHWs were local residents employed by two local not-for-profit organization—Red Cross and Genadendal Legal Information Desk. All CHWs in the Genadendal/Greyton area (*n* = 22) were invited to participate in the study through their organization’s administrative office. Two CHWs (one male and one female) who participated in the training did not interview for personal and family reasons. The final study sample consisted of 20 CHWs, yielding a response rate of 90%. The inclusion criteria were: CHWs actively working at least 20 h per week and willing to participate in interviews. Participants were not financially incentivized to participate, although they were provided with light refreshments during the interview and discussion. Demographic information on participants is presented in Table 2.

### 2.3. Data Collection and Analysis

Twenty in-depth interviews were conducted with community health workers following their completion of a two-week self-management training. All interviews were conducted by the first author at places mutually agreed upon with participants, such as their workplaces or in the car during their home visits. All participants received information on the academic nature of the research and its objectives, provided consent to participate in the study and gave permission for audio recording. Interviews lasted 30 min on average. Data collection continued until data saturation was attained [23].

Audiotaped data were transcribed, reviewed for correctness and read by the research team to familiarize themselves with the interview content and develop initial codes. Four researchers crosschecked initial codes and analyzed and coded all transcripts. A thematic analysis was conducted along the guidelines proposed by Braun and Clarke [24]. Transcripts and codes were entered into NVivo 12 (QSR International, Burlington, MA, USA) for further analysis. As the narratives of CHWs’ work environment was examined more closely, findings emerged around common themes of intrinsic and extrinsic challenges CHWs face with performing their roles.

## 3. Results

The majority of the participating CHWs were female (95%), with a mean of 8.3 years (range = 3–18 years) of community health work experience. All CHWs lived either in the communities they served or in neighboring communities. They identified multiple role functions such as serving as a screener (identifying client problems and referring appropriately), educator (conducting patient and family education), trainer (providing the clients or family with skills for them to be physically active), health promoter, and basic medical care provider.

### 3.1. Emerging Themes

**Figure 1:**
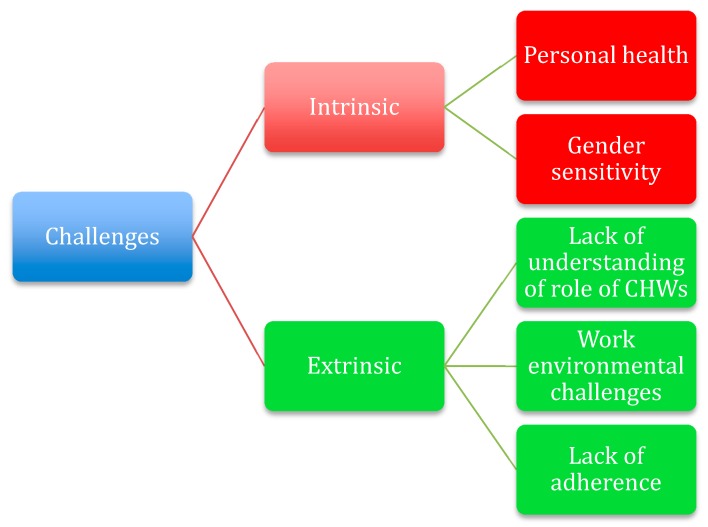
Mapping of challenges faced by CHWs within Greyton and Genandendal communities, Western Cape, South Africa.

Based on data, two main challenge themes (a) intrinsic factors (e.g., CHWs coping with their own emotional, physical, and psychological challenges and gender sensitivity challenges) and (b) extrinsic factors (e.g., lack of understanding of the CHW role by various stakeholders; environmental challenges: lack of resources in terms of medical supplies, lack of transport; lack of patient and family adherence) emerged (Figure 1).

### 3.2. Intrinsic Factors

#### 3.2.1. Personal Health and Emotional Challenges

Personal health and emotional challenges identified by the participants included challenges with their own physical health but also the emotional and psychological impact of the work. Many CHWs had chronic conditions themselves and they had direct personal experience of economic, social and emotional abuse.

*“My health is difficult because I am most of the time at the hospital and at clinics because of my back [back pain]”* (CHW327_001)

Participants expressed how working with an individual with a terminal illness affected them.

*“Especially the cancer patients. I have worked with one and it was very traumatic for me, you see, because her whole body was full of sores”.* (CHW328_007)

*“I got so emotional, I am crying and as I washed that guy, I was afraid of the cancer. Yeah, the skin cancer. Yeah, and you must touch, you must work with that patient. It was dirty skin and is cracking and bleeding”.* (CHW328_008)

*“We can’t do a lot about it but maybe you can talk to someone like a counselor or somebody, but is very difficult”.* (CHW330_002)

Participants expressed challenges within their homes, managing familial relationships and health conditions of family members which further affected them emotionally and could hinder their work performance.

*“I have been through a lot of things. My husband left me with five boys and a divorce, and he was living in the house with us with the other [new] wife and a child. So my children and I went through a lot that time”.* (CHW328_007)

*“My husband is at home, sick. He has a heart condition that can’t be fixed and he is doing okay now, but that is very stressful for me because I go out in the community. I have to worry about him all the time while I’m out because he’s got a lot of pain”.* (CHW328_004)

*“I must work because why husband had a stroke and there is no income in the house”.* (CHW328_008)

The need for self-care was described as important.

*“We have to be prepared 24/7, which means whatever happens in case of emergencies you have to be available. It doesn’t matter what time, you know. Now, see, for me it’s a difficulty because of my children… You get so into this job that sometimes you forget about yourself. It’s all about other people, about the job, about going back to the house, about the kids, but at the end of the day you must also be looked after”.* (CHW329_001)

#### 3.2.2. Gender Sensitivity

Gender sensitivity was also highlighted by the participants. The only male participant articulated the challenges that he experienced, indicating that this was a female-driven profession.

*“Because I am a man it’s always difficult. People like for instance, not much male patients or female patients is comfortable with me and also I think because I’m young it’s much more difficult for them to accept me because the view they have is that it’s a woman’s job”.* (CHW329_003)

On the other hand, the female CHWs indicated that information relevant to men was not received well by male patients if given by a female CHW, as male patients did not think that female CHWs understood men’s challenges clearly.

*“The medical men circumcision, that topic is difficult because we [women] have to go out there and talk to men and they target us for …lack of experience on this topic”.* (CHW330_003)

### 3.3. Extrinsic Factors

#### 3.3.1. Lack of understanding or appreciation of the role of CHWs

Lack of understanding or appreciation of the role of CHWs by various stakeholders was highlighted by the participants. For example, CHWs sometimes struggle with access to the patients’ homes as well as patients being non-receptive to CHWs. A greater expectation for services rendered without recognition for those who provide the services was also expressed.

*“Sometimes people let you in; sometimes people don’t want you in their home”.* (CHW327_004)

*“Some health providers, they just want more and more from us, but they are not seeing or recognizing us for our services to the community”.* (CHW330_003)

*Now you come in your area especially and some people will tell you “Why do you have to ask me those questions again because that nurse was already here.” Some people are very moody sometimes, especially when the weather is not so good in the winter. They will tell us “What are you doing outside? It’s raining,” and “We want to sleep”.* (CHW328_001)

#### 3.3.2. Workplaces

Workplaces play an important role in how employees manage their own health and influence the health behaviors of their coworkers. Deliberating further on workplace challenges, participants recounted many work environment barriers. These included lack of appropriate medical supplies for handling certain types of health conditions, lack of transportation for workers and for patients, long work hours, and poor compensation. Lack of transportation for patients impacted treatment adherence in a negative way.

*“There’s sometimes a long distance that we must walk, a long distance, and sometimes the clinic don’t have the medication…”.* (CHW329_004)

*“One sore was in the back of her, and you can put your whole fist into it. That kind of thing it really stressed me out because she didn’t have the things [medical equipment] that could make it easier for her. There are so many things that were very tragic and hard for me to see because her whole body was full of sores. I have to turn, I must lift her up and I must put her on the tummy so I can do the back, and while I was doing it she was crying the whole time”.* (CHW328_007)

#### 3.3.3. Lack of Adherence

Lack of adherence was mentioned several times by CHWs. They felt that their patients required constant monitoring in order to ensure that medications were administered in a timely manner, whether by themselves or family members. They also indicated that when patients were referred to clinics for further health management, these patients were not inclined to visit these clinics. Although this challenge can also be linked to inadequate transportation services, other factors hinge on socio-cultural influences, health literacy, and personal attitudes as contributing factors.

*“Some patients struggle to get to the clinic. The problem is with transport and so patients don’t get their medication”.* (CHW329_006)

*“That patients, most of the time difficulties is their tablets. They don’t drink the tablets that I (give them), ...for example, the epilepsy patient, she didn’t drink the tablets right …”* CHW327_001

*“Did you eat, did you take your medicine? We have to go in there continuously to see if they are doing it”.* (CHW327_003)

*“At first it was we must get women to go for Pap smears. They didn’t want to go. We had to explain (that) it’s for their own good, it’s for their own health. At last they agreed and did go to the clinic for Pap smears. Many of them were scared, they didn’t know how a Pap smear was done”.* (CHW328_002)

Lack of treatment adherence was noted even among CHWs themselves, as one pointed out,

*“I’ve got some health issues myself. My action plan is to work on that, to take my medication. I don’t like medication, …the second day I forgot to do medication…. so, my plan is to finish my medication on time, drink it every day.”* (CHW328_002)

## 4. Discussion

Understanding challenges faced by CHWs is essential in order to ensure the delivery of stellar health services by these vital health care providers. The results of this study show that the challenges faced by community health workers are both intrinsic and extrinsic.

There is a definite need to address the intrinsic needs of CHWs in order to ensure that their emotional and physical well-being is improved. CHWs reported both emotional and physical challenges from managing their own chronic health conditions and that of family members and clients. The CHWs present with chronic conditions themselves, which not only affect work attendance but also their ability to cope with the rigors of the job such as walking long distances to patients’ homes, as well as lifting, bathing, and transferring patients. Because CHWs receive less in-depth training on how to manage the degeneration of chronic health conditions (for themselves and clients), they may have reduced efficacy to manage job-related stresses alongside managing their own health [13]. This suggests that CHWs may be at increased risks of side effects of job-related stressors including mental and emotional distress. Caregivers often report a lower level of quality of life and higher levels of stress than non-caregivers [25,26,27,28]. Our study explored how the health status of patients and close family members (husband, wife, parents or children) had an emotional impact on CHWs.

The poor socio-economic conditions of patients—limited access to health care, lack of financial resources, and cost of medication—play an important role in influencing medication adherence. CHWs acknowledged that their patients do not adhere to their medical prescriptions mainly for financial reasons like lacking transportation to go and refill their prescriptions, and where medication requires eating first before taking it, some patients lacked the money to buy food. Poor treatment adherence demotivates CHWs, because their labor of love gets eroded as the health of their patients fail to improve. According to the World Health Organization, people who have social support from family, friends, or caregivers to assist with medication regimens have better adherence to treatment [29]. For most patients in the study, CHWs are the only friends or “family” that they have.

Our study revealed that patient attitudes toward male CHWs were sometimes negative, as the CHW role is often viewed as a “female” profession [15]. Although only one male CHW participated in our study, viewing the CHW role as a female responsibility may undermine male CHWs’ ability to engage with patients and may compromise their work-related emotional health [30]. Although our data seem to suggest that female CHWs were better received by the community, some conditions occurring only in male patients, such as medical male circumcision, were reportedly more difficult for female CHWs to manage due to patient attitudes. This suggests that the challenges CHWs face are in part gender sensitive. Although there are fewer male CHWs compared to female CHWs, it appears that aligning CHWs roles to the gender of their patient may improve health outcomes for the patient and increase CHW job satisfaction.

In addition to these intrinsic factors, certain extrinsic factors also affect CHWs’ ability to optimize their service delivery. Our findings regarding insufficient transportation and lack of medical equipment and other resources are similar to other studies which also highlighted lack of supplies and transport as challenges [15]. Inadequate transportation is also a common problem in community-based care [31,32]. Although the ideal is that CHWs provide a service in an area that is within walking distance, the reality is that they often cover much larger areas [31], which is further complicated by poor weather conditions such as rain and heat. Lack of health resources also constrains CHW’s effectiveness. CHWs interviewed in the current study noted that a lack of equipment often meant that patients had to travel to the nearest health center for services that CHWs could have provided in the patient’s own community. The lack of stock or supplies available for CHWs could mean that staff have to make additional trips to health care centers, using scarce clinical time for these administrative functions [31]. The need to travel to health care centers has a financial burden on both patients and CHWs, as they often have to finance their transportation to access such facilities. Lack of needed equipment and medical stocks create barriers to care and may increase clinical inefficiencies. Additionally, participants in this study reported lack of recognition for their work. The CHWs expressed a lack of appreciation of their role by their patients and community members they serve. Although community health workers play a vital role in the primary health care model, lack of recognition may lead to lower morale and increased turnover, placing even more burden on an already strained rural health system [32].

## 5. Implications for Practice

Because CHWs serve as critical “links” and “bridges” between their communities and health care providers [33,34,35], there is a need to improve personal health, workplace and social conditions for CHWs. Interventions could include self-management training, motivational interviewing, support groups for CHWs and their patients, and continued education for new and current CHWs. We have attempted to show here and elsewhere [13] that the effectiveness of CHWs may be constrained by factors intrinsic and extrinsic to CHWs.

CHWs have increasingly become an important part of the health care system and the population they serve has become more diverse with respect to age, sexual orientation, race, and socio-economic status. Despite the increased reliance on CHWs to address the needs of diverse patient populations, research has shown that CHWs are not effectively trained or retained [36]. Therefore, there is need for educational programs targeted at CHWs, patients, and community members (including spouses of CHWs) to educate them on the role of CHWs.

Rural CHWs tend to be from low-income households, have limited educational training, and may experience poor health themselves. There is a need to include coping strategies in training programs for individuals with, and when caring for patients with, chronic conditions.

Rural CHWs are a key community resource—as such, community support is paramount for effective service delivery. CHW programs can engage communities and build a pool of volunteers to help with ride sharing for CHWs and/or patients and with general support of patients where possible (with patient consent). Communities can also establish food banks targeted at helping patients who are not able to purchase enough food for themselves. Along the same vein, community organizations can establish low-cost recognition and appreciation programs for CHWs. Such opportunities have the potential to boost morale among CHWs and their patients.

## 6. Limitations

Several aspects of the study may limit the generalizability of findings. This study was conducted in two rural communities with relatively longstanding CHW programs and may be less applicable in settings in which health resources are more broadly available. Results may also reflect the largely female CHW workforce—targeted study of male CHWs may reveal additional perspectives. Findings may be limited in that they emerge from a broader study focusing on the evaluation of a training program. Finally, our study communities have strong ties to the research team and may reflect a candor and comfort level when discussing difficult aspects of the work that may not emerge as easily among samples with few or no ties to the research team.

## 7. Conclusions

This study addressed one aspect of the Two-Factor theory, namely the challenges that CHWs experience in their work. If such intrinsic and extrinsic challenges are mitigated by focused programmatic efforts, emphasis can shift to addressing motivational factors that enhance workers’ experience in their CHW roles. The engagement of CHWs and their communities is critical to the process of improving the health of CHWs and the quality of care they provide to community members. Empowering CHWs by raising awareness of their challenges and motivational factors can help to engage them as active partners in the formulation of patient-centered education and training which takes into account the needs of CHWs as well as the context.

## Figures and Tables

**Table 1:** Semi-structured interview guide for community health workers.

Interview Questions
Please tell me more about yourself: how long you have lived in this community, what you do as a community health worker (CHW), and how long you have done that? Thinking back before the self-management training, what are some of the challenges/difficulties you faced in: Promoting the help of your patients? Promoting your own help? Thinking of the self-management training you had last week: What made you want to sign up for the program? What are some of the important things you got from the training? Looking ahead, do you think the skills you gained will help with your job? Explain how you see yourself using the skills you gained from the training. Patients/clients Self Community (broader dissemination) Do you feel empowered by this program? Explain What other support do you need to use the skills you gained from the training? What would motivate community members to come to this training? What would help participants implement their action plans and be successful? Was the training well delivered? What could be improved to help participants like you learn the skills better? curriculum/content class structure delivery Is there anything else you would want to say about the program?

**Table 2:** Participant socio-demographic information and health characteristics of participants.

Participant Socio-Demographic Information (*n* = 20 *)	*n* (%)
Gender Male * Female	1 (5) 19 (95)
Race Colored (per self-designation)	20 (100)
Hours worked per week <20 20–29 30–39 40+	3 (15) 10 (50) 2 (10) 5 (25)
Education status Some high school or less Finished high school or more	14 (70) 6 (30)
Age (years, mean (SD))	40.0 (10)
Employment duration as CHW (years, mean (SD))	7.27 (2.62)
Health Characteristics of Participants (*n* = 20 *)	***n* (%)**
In the past week, how often has your health interfered with your work life? Always Usually Sometimes Rarely/never	5 (25) 4 (20) 10 (50) 1 (5)
In the past week, how often has your health interfered with your personal life? Always Usually Sometimes Rarely/never	9 (45) 5 (25) 6 (30) 0 (0)
Did you have any difficulties with your daily activities because of your health in the past week? Always Usually Sometimes Rarely/never	9 (45) 0 (0) 10 (50) 1 (5)
In general, how would you say your health has been in the past week? Excellent Very good Good Fair/poor	3 (15) 1 (5) 14 (70) 2 (10)

* Excludes two CHWs who did not interview. CHW—community health worker; SD—standard deviation.
